# Surface Chemistry of Polymers

**DOI:** 10.3390/polym12112757

**Published:** 2020-11-23

**Authors:** Alenka Vesel

**Affiliations:** Department of Surface Engineering, Jozef Stefan Institute, Jamova cesta 39, 1000 Ljubljana, Slovenia; alenka.vesel@guest.arnes.si

Surface chemistry plays a key role in modern applications of polymer materials. Polymers exhibit moderate surface properties; therefore, they do often not meet the desired criteria for particular applications. The polymer materials should often be pre-treated with chemical substances to achieve good adhesion of various coatings, appropriate interaction with living tissues, cells or biological liquids, the ability to bond or transmit various gases, and the ability to release substances that have been captured within the polymer structure. Various surface chemistries should be employed to meet these criteria. The polymer surfaces can be treated with, either liquid or gaseous reactants. In both cases, the functional groups on the polymer surfaces change their properties, either reversibly or irreversibly. The liquid treatments are usually rapid. Therefore, they are preferred in practical applications as long as such treatments do not represent an ecological or health hazard. Otherwise, it is better to perform gaseous treatments by application of non-equilibrium gaseous plasma, which consists of various species that are reactive enough at room temperature. Once the surface is functionalized with the appropriate groups, further treatments can be applied to obtain the desired surface finish. 

The contributions in this special issue were carefully selected to give a comprehensive, but focused insight in both pre-treatments of polymer surfaces prior to further treatments, and specific applications of such treatments. This special issue contains a couple of review papers on pretreatment of polymer materials to obtain the desired hydrophilicity, including rarely tackled phenomenon on surface activation of fluorinated polymers. 

A review on the plasma-stimulated super-hydrophilic surface finish has been published in this special issue [1]. A couple of mechanisms leading to super-hydrophilicity of polymers have been reviewed. A one-step exposure to reactive plasma is the simplest approach. It actually enables super-hydrophilicity of a few aromatic polymers, but does not assure such surface finish of most other polymers. The reason is the inability of such plasma treatment to assure both appropriate morphology and functionalization with polar functional groups. An enhanced approach is the exposure of a polymer to reactive gaseous plasma and simultaneous deposition of etching inhibitors. Such treatment is typically performed using oxygen plasma sustained by capacitively coupled discharges where the powered electrode is subject to bombardment by positively charged ions, which causes sputtering. A further alternative is a deposition of nano-coating on the polymer material, followed by deposition of a very thin film of a material that can be made super-hydrophilic by a brief functionalization with oxygen plasma. 

Upper mentioned techniques fail in the case of fluorinated polymers. A review of surface activation of polytetrafluoroethylene (PTFE) by gaseous plasma was prepared by Primc [2]. The author pointed out that the usual technique for making polymers super-hydrophilic, i.e., treatment with oxygen plasma, is obsolete because the interaction leads to etching of such materials instead of functionalization. A correlation between the water contact angle (WCA) and the concentration of oxygen in gaseous plasma revealed that highly hydrophilic surfaces were reported only when other gases were used, and oxygen was only present as a gaseous impurity. The only known technique that enables the super-hydrophilic surface finish of PTFE is the application of gaseous plasma with an appropriate concentration of ammonia and water vapor. Such a gaseous plasma enables both etching (and thus nanostructuring of the fluorinated polymer) and functionalization with polar functional groups. The super-hydrophilic effect was explained by bond scission caused by irradiation with vacuum ultraviolet (VUV) photons and surface chemistry caused by the interaction with NH_x_ radicals from the gas phase. 

The effect of VUV radiation from gaseous plasma is not only beneficial for hydrophilization of fluorinated polymers but also for modification of surface structure and composition as elaborated in the paper by Zaplotnik et al. [3]. The authors performed systematic research on the influence of VUV radiation arising from atmospheric pressure plasma jet (APPJ) sustained in Ar gas on the surface properties of polystyrene (PS) polymer. The penetration depth of VUV is rather short because of its high absorption probability; therefore, the chemical modifications are concentrated on the surface film, which can be probed by various techniques, including X-ray photoelectron spectroscopy (XPS). The authors compared the surface finish, obtained by treatment with VUV photons only, or APPJ jet, and found much better functionalization using radiation only. Such an effect was explained by the lack of exothermic chemical reactions that are likely to occur on the surface of polymers upon treatment with gaseous plasma. 

Low-temperature plasma modification was also investigated by using a kinetic approach [4]. The model polymer was styrene-butadiene copolymer, and the aim was to improve the wettability and, thus, the adhesion of different coatings. Low-pressure gaseous plasma was sustained in various gases, both chemically inert and highly reactive. The effect of plasma treatment on the surface finish revealed a range of optimal conditions. For short treatment times, the peel strength increased rapidly but stabilized and decreased with further treatment. Theoretical predictions were supported with experimental results. An excellent correlation between the concentration of OH groups on the copolymer surface and the peel strength was observed. The required treatment time for the optimal surface finishes was about 1 s at the discharge power of the order of 10 W. 

The adhesion of coatings on the plasma-treated polymer surface was also addressed by Potrč et al. [5]. Polyethylene (PET) or polypropylene (PP) films were treated in a flowing afterglow. The afterglow is free from charged particles but rich in neutral atoms which are capable of chemical interaction with polymer surfaces. The surface of both polymers became saturated after about a minute of treatment with the oxygen afterglow. Such surface finish was found an appropriate for the deposition of the bioactive coatings. Different solutions of chitosan biopolymer were prepared and applied to plasma pretreated polymer foils to suppress the oxygen permeability, and stimulate the antimicrobial activity. The technique is useful for application in food packaging. 

Gaseous plasma pretreatment of PP was also found useful for suppression of interaction between specific compounds of human blood and Eppendorf tubes [6]. The Eppendorf tubes were treated with atmospheric-pressure plasma jet using Ar as the processing gas. Significant differences between different types of Eppendorf tubes made from the same material have been observed. Even untreated Eppendorf tubes from some suppliers already contained approximately 10% of oxygen, indicating different production technology. Plasma treated Eppendorf tubes were incubated with human blood, and the irreversible adhesion of the extracellular vesicles was probed by the flow cytometry. The results were rather scattered, but in all cases, a significant improvement in the hemocompatibility of the surface of Eppendorf tubes was observed. 

As elaborated in the above-cited papers, gaseous plasma treatment always causes surface functionalization and usually also some etching. Non-equilibrium gaseous plasma, however, could also be used as a medium for deposition of various coatings. Kousal et al. [7] used low-power gaseous plasma for depositing thin polymer films on different substrates. Polyethylene oxide (PEO) of various molecular weights was mounted into the reaction chamber. The experimental system was heated to elevated temperatures to stimulate evaporation upon vacuum conditions. The vapors partially decomposed upon plasma conditions and adhered on the substrate to form a polymer-like film. The coatings were found semi-transparent for peptide nisin. The technique is attractive for application in pharmacy as a method for controlled drug release. The nisin release kinetics from the PEO-like film was found rather tunable. The release of this chemical compound upon soaking in water was found to depend significantly on the deposition conditions, and in particular, on the discharge power and temperature of the precursor. 

Polyethylene terephthalate (PET) is sometimes used as a material for body implants such as vascular grafts and polymeric stents. Fras et al. [8] studied the adsorption of sulfated xylans on PET polymers. No plasma pretreatment was performed, but the polymer was carefully cleaned. The xylan solutions were prepared in distilled water and carefully adjusted pH. Deprotonated anionic groups in the solution enabled appropriate surface chemistry, so almost perfect stoichiometry between the ammonium, sulfate, and carboxyl groups was obtained. Adsorption kinetics were investigated by quartz crystal microbalance (QCM), which showed the formation of viscoelastic films. Excellent stability, hydrophilicity, and anti-thrombogenic properties were found for such materials, which represents one of the most promising biocompatible coatings for vascular implants.

Polymer materials are also used in environmental engineering and biotechnological processes. Interaction between yeast cells and the model surfaces was elaborated by Botelho et al. [9]. Adhesion and immobilization of *Yarrowia lipolytica* on residual plastics were investigated using various substrates such as PET, PTFE and polystyrene (PS). Good adhesion to different polymer surfaces was observed in a broad range of pH values of the culturing medium. The authors found that cell extracts, treated in ultrasonic baths maintained their enzymatic activity and could be attached even to the PET surface, and thus, used as immobilized biocatalyzers. 

An alternative to polymer functionalization is the application of surfactants. Various surfactants have been employed for investigation of the interaction with a drag-reducing polymer (sodium carboxymethyl cellulose) by Yang and Pal [10]. The interaction was found shear-thinning in nature and had a strong effect on the consistency index of the surfactant solution. The interaction was studied by measuring surface tensions at different surfactant concentrations. The systematic measurement showed strong interactions between anionic surfactants and an anionic polymer surface, while the interaction between the anionic polymer and non-ionic surfactant was moderate.
